# Improved calculation of warming-equivalent emissions for short-lived climate pollutants

**DOI:** 10.1038/s41612-019-0086-4

**Published:** 2019-09-04

**Authors:** Michelle Cain, John Lynch, Myles R. Allen, Jan S. Fuglestvedt, David J. Frame, Adrian H Macey

**Affiliations:** 1 Environmental Change Institute, School of Geography and the Environment, University of Oxford, South Parks Road, Oxford, OX1 3QY UK; 2 Oxford Martin School, University of Oxford, 34 Broad Street, Oxford, OX1 3BD UK; 3 Atmospheric Oceanic and Planetary Physics, Department of Physics, University of Oxford, Parks Road, Oxford, OX1 3PU UK; 4 Center for International Climate and Environmental Research (CICERO), PO Box 1129 Blindern, 0318 Oslo, Norway; 5 New Zealand Climate Change Research Institute, Victoria University of Wellington, PO Box 600, Wellington, New Zealand; 6 Institute for Governance and Policy Studies, Victoria University of Wellington, PO Box 600, Wellington, New Zealand; 7 Institut d’Etudes Avancées de Nantes, 5, Allée Jacques Berque, 44000 Nantes, France

**Keywords:** Climate-change mitigation, Climate and Earth system modelling, Climate-change mitigation, Climate-change policy

## Abstract

Anthropogenic global warming at a given time is largely determined by the cumulative total emissions (or stock) of long-lived climate pollutants (LLCPs), predominantly carbon dioxide (CO_2_), and the emission rates (or flow) of short-lived climate pollutants (SLCPs) immediately prior to that time. Under the United Nations Framework Convention on Climate Change (UNFCCC), reporting of greenhouse gas emissions has been standardised in terms of CO_2_-equivalent (CO_2_-e) emissions using Global Warming Potentials (GWP) over 100-years, but the conventional usage of GWP does not adequately capture the different behaviours of LLCPs and SLCPs, or their impact on global mean surface temperature. An alternative usage of GWP, denoted GWP*, overcomes this problem by equating an increase in the emission rate of an SLCP with a one-off “pulse” emission of CO_2_. We show that this approach, while an improvement on the conventional usage, slightly underestimates the impact of recent increases in SLCP emissions on current rates of warming because the climate does not respond instantaneously to radiative forcing. We resolve this with a modification of the GWP* definition, which incorporates a term for each of the short-timescale and long-timescale climate responses to changes in radiative forcing. The amended version allows “CO_2_-warming-equivalent” (CO_2_-we) emissions to be calculated directly from reported emissions. Thus SLCPs can be incorporated directly into carbon budgets consistent with long-term temperature goals, because every unit of CO_2_-we emitted generates approximately the same amount of warming, whether it is emitted as a SLCP or a LLCP. This is not the case for conventionally derived CO_2_-e.

## Climate mitigation: new metric for comparing greenhouse gases

Effective climate policy necessitates being able to compare the impact of different greenhouse gases on warming. This is commonly accomplished by converting non-CO_2_ emissions to CO_2_ equivalent emissions using Global Warming Potentials (GWP). However, the conventional GWP approach masks the true behaviour of short-lived climate pollutants (SLCPs) and an alternative GWP approach has recently been developed. Michelle Cain of the University of Oxford and a team of international colleagues extend this alternative GWP approach that slightly underestimates the impact of SLCPs on warming. They develop a new metric that approximates both the short-timescale climate response to changing SLCP emission rates and the long-timescale adjustment of the climate system to past emissions. It is demonstrated that methane (CH_4_) emissions can be converted to a CO_2_ equivalent emissions in a way that best represents the temperature impacts of methane emissions. The new metric offers a way to assess the relative merits of climate policies in a manner consistent with the Paris Agreement.

## Introduction

Comprehensive climate policies must appraise a range of greenhouse gases and aerosols, which can differ significantly in their radiative efficiencies and atmospheric lifespans, and hence the nature of their climate impacts.^1^ To reflect this, different climate pollutants are often expressed using a common emission metric. Emissions reporting under the United Nations Framework Convention on Climate Change (UNFCCC) now requires the use of 100-year Global Warming Potential (GWP_100_) to account for all gases as carbon dioxide equivalent (CO_2_-e) quantities. Despite its prevalence in the UNFCCC and national climate policies, GWP has received criticism,^2–4^ not least that it cannot be used to appraise temperature-related goals,^5^ and other equivalence metrics have been proposed.^6–9^ Indeed, Shine^3^ notes that strong caveats were in place when GWP was introduced in the Intergovernmental Panel on Climate Change’s First Assessment Report^10^: “It must be stressed that there is no universally accepted methodology for combining all the relevant factors into a single [metric]… A simple approach [i.e., the GWP] has been adopted here to illustrate the difficulties inherent in the concept.” Working Group 1 of the Fifth Assessment Report, AR5, did not recommend any metric and emphasised that the choice of metric depends on the specific goal of the climate policy. In AR4, however, the GWPs were the recommended metric to compare the effects of long-lived greenhouse gases,^11^ and AR5 values of GWP_100_ have now been adopted for emissions reporting (see the textual proposal from 12 December 2018 on the transparency framework for action and support referred to in Article 13 of the Paris Agreement: https://unfccc.int/process/bodies/subsidiary-bodies/ad-hoc-working-group-on-the-paris-agreement-apa/information-on-apa-agenda-item-5).

The temperature response to emissions is ambiguous under GWP^1,12,13^ and this ambiguity is particularly relevant in the context of the Paris Agreement, given its stated aim of ‘holding the increase in the global average temperature well below 2 °C above pre-industrial levels and pursuing efforts to limit the temperature increase to 1.5 °C.’ Beyond the reference to a balance of emissions by sources and removals by sinks well before the end of the century, neither the means by which this is to be achieved nor the metrics used to assess progress are explicitly stated.^14^ Tanaka and O’Neill^15^ demonstrate that net-zero aggregate CO_2_-e emissions based on GWP_100_ (which is often assumed to be the definition of the balance of sources and sinks described in the Paris Agreement) are not essential to limit warming to 1.5 °C. Wigley^16^ posits that the balance of sources and sinks in Article 4.1 of the Paris Agreement is scientifically inconsistent with the temperature goals in Article 2.1. These papers show how moving from the temperature goals articulated in the Paris Agreement to emissions targets and profiles is not something that is currently well-handled by conventional carbon accounting; they also show that the area is receiving renewed scrutiny as countries, firms and sectoral bodies try to work out mitigation strategies of their own.

This paper demonstrates a method that unambiguously links aggregated greenhouse gas emissions with their warming outcomes on decade to century timescales, allowing short-lived climate pollutants (SLCPs) to be brought into a carbon budget framework.^17^ It is designed to be useful for informing policies that specifically aim to limit global warming, as is required under the Paris Agreement. This method builds on the revised usage of GWP, denoted GWP*, proposed in Allen et al.,^12,18^ building on Shine, et al.^6^ Specifically, we address a shortcoming in the originally proposed definition of GWP*, in that it did not account for the delayed temperature response to past increases in SLCP emissions, bringing aggregate emissions into closer agreement with both CO_2_-forcing-equivalent emissions^19^ and the temperature response.

## Results

### A revised definition of GWP*

A new usage of GWPs, denoted GWP*, allows emissions of short-lived and long-lived climate pollutants (SLCP & LLCPs) to be more consistently expressed within a single metric by equating a change in the emission rate of an SLCP as equivalent to a single emissions pulse of a long-lived pollutant. As originally defined in Allen, et al.,^18^ a step-change in emission rate of an SLCP (Δ*E*_SLCP_ tonnes per year) is equivalent to a one-off pulse emission of Δ*E*_SLCP_ × GWP_*H*_ × *H* tonnes of CO_2_, where GWP_*H*_ is the conventional Global Warming Potential relative to CO_2_, integrated over a time-horizon *H* years. Emissions of LLCPs, defined here as those having an atmospheric lifetime longer than *H*, will still behave as a cumulative pollutant within time-horizon *H*, and therefore equivalent emissions for LLCPs are derived simply by multiplying those emissions by GWP_*H*_.

This rate-based equivalence for SLCPs overcomes the problems inherent in GWP (or any pulse-based metric) in not adequately distinguishing their largely non-cumulative behaviour. However, although a sustained SLCP emissions rate will result in a stable atmospheric concentration and hence maintain the same level of forcing, some additional long-term warming will occur while the climate system is still equilibrating to past increases in SLCP emissions. Note this is not a cumulative impact of emissions mirroring that of CO_2_: it is, rather, the delayed response associated with equilibration to a past increase in forcing. After a sufficiently long period of constant emissions (on the order of centuries), SLCP-induced warming will stabilise, whereas CO_2_-induced warming continues to increase as long as CO_2_ emissions remain above zero. After CO_2_ emissions reach zero, ongoing thermal adjustment in surface temperature is largely balanced by ocean uptake of CO_2_,^20^ at least in the absence of strong Earth System feedbacks.^21^ The multi-century component of the thermal response of the climate system, together with carbon cycle feedbacks,^22^ act to prolong the warming impact of SLCP emissions.^23^ As noted in Allen, et al.,^12^ this can be incorporated ‘by including a small contribution that scales with time-integrated [SLCP] emissions.’

This component was not pursued in Allen, et al.^12^ for simplicity and because the contribution of this multi-century adjustment to past increases in SLCP emissions is small compared to the impact of current changes in SLCP emissions under the scenarios considered in that paper (see their Fig. 2 and supplementary Fig. 1 to this paper). Nevertheless, it may be significant for individual countries whose SLCP emissions have increased within the past half-century or so and are now approximately stable.

Thus, we propose a re-definition of GWP* to incorporate both timescales, as well as providing a theoretical justification below, we here adjust this empirically to produce the best fit between cumulative CO_2_-warming-equivalent (CO_2_-we) emissions and resultant warming (see below and Methods for full details). Calculated using this re-defined GWP*, CO_2_-we emissions of an SLCP in a given year are defined:

1 $$E_{{\mathrm{CO}}_2{\mathrm{we}}} = {\mathrm{GWP}}_H \times \left[ {r \times \frac{{\Delta E_{{\mathrm{SLCP}}}}}{{\Delta t}} \times H + s \times E_{{\mathrm{SLCP}}}} \right]$$

where GWP_*H*_ is the conventional global warming potential for a given SLCP over time-horizon *H*, Δ*E*_SLCP_ the change in SLCP emission rate over the preceding Δ*t* years, *E*_SLCP_ the SLCP emissions for that year, and *r* and *s* the weights assigned to the rate and stock contributions, respectively. The only difference between this formulation and that of Allen et al.^12,18^ is that they used *r* = 1 and *s* = 0. Including the time period Δ*t* spreads the CO_2_-we pulse corresponding to a change in SLCP emission rate over Δ*t*. Allen et al.^12^ suggest at least 20 years, which has the effect of reducing the volatility in CO_2_-we emissions and improving the correspondence with temperature response. The first (“rate”) term on the right-hand side, $r \times \frac{{\Delta E_{{\mathrm{SLCP}}}}}{{\Delta t}} \times H \times {\mathrm{GWP}}_H$, represents the response to the changing SLCP emission rates. The second (“stock”) term, *s* × *E*_SLCP_ × GWP_*H*_, is added to represent the long-term equilibration to past increases in forcing, which can be approximated by a small term scaling with cumulative SLCP emissions. In other words, the rate term approximates the short-timescale climate response to a change in radiative forcing; the stock term approximates the long-timescale equilibration which occurs even when there is constant radiative forcing.

The exact values of *r* and *s* will depend on the precise timescales of the climate response to radiative forcing, on how long ago the increase in SLCP emissions occurred, and on carbon cycle feedbacks, all of which are uncertain and scenario-dependent. Constraining *r* + *s* = 1 ensures that total CO_2_-we emissions over 100 years corresponding to a steady SLCP emission starting in year 1 are the same as total CO_2_-e emissions would be, consistent with the original derivation of GWP* presented in Allen, et al.^18^ The ratio *s*/(*rH*) corresponds to the fractional rate of decline of SLCP emissions for these to be considered equivalent to a zero rate of CO_2_-we emissions and hence not cause further warming. This depends on the SLCP and details of the scenario in question. Our objective is a simple and reliable indicator of the relationship between emissions and recent and near-term future warming associated with the largest non-CO_2_ climate drivers, so we estimate *r* and *s* using a multiple linear regression onto the response to methane emissions in commonly used scenarios (representative concentration pathways, RCPs), focusing on the time period 1900–2100.

The method is applicable to other SLCPs, but the optimal values of *r* and *s* could be different for each SLCP dependent on past emissions. As many short-lived industrial gases have only started being released in recent decades, the warming responses to these gases is likely distinct to those for methane, with a greater emphasis on the immediate effects of changing emission rates, and thus not necessarily reflecting the same *r* and *s* values as derived for methane here. That said, GWP*, like any metric, depends on strong assumptions of linearity, so the additional precision may not be worth the additional complexity. The application of GWP* to specific other gases is beyond the scope of this paper but warrants further investigation, given their impacts are substantial and potentially growing. For example, the radiative forcing from total halocarbons (long and short-lived) is less than half that from methane^1^ but may grow with increasing demand for air conditioning.

### Empirical estimation of flow and stock contributions

The global mean surface temperature (GMST) responses (ΔT) to methane radiative forcing from the RCP2.6, 4.5 and 6 scenarios taken from the AR5 database,^24^ were derived using the default configuration of the FaIR simple climate model.^25^ Methane was chosen as it has the largest impact on radiative forcing of all the SLCPs, and these scenarios were used as they represent pathways approximately in line with current policies, as well as a more ambitious pathway (RCP 2.6). Multiple linear regression was then used to find values of *r* and *s* to best fit the relationship between cumulative emissions of CO_2_-we and Δ*T* (using the RCP emissions from the AR5 database), constrained such that *r* + *s* = 1. Using this approach, *r* = 0.75 and *s* = 0.25 are the mean values based on the three RCPs and are found to provide a good fit for all three (see methods section below).

**Fig. 1:**
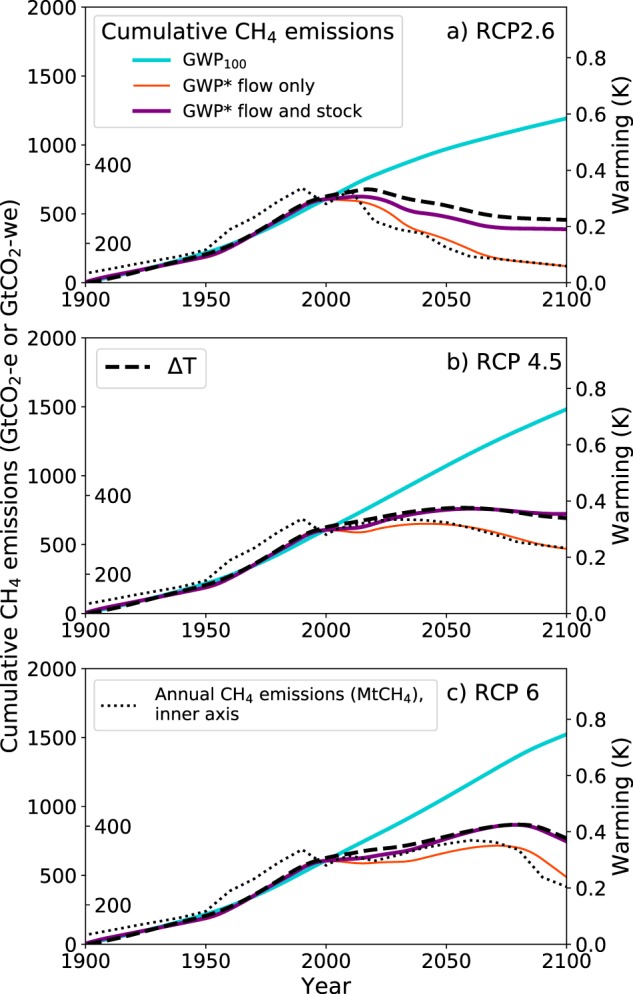
Cumulative methane emissions (left axis) from historical data since 1900 combined with RCP 2.6 (upper), RCP 4.5 (middle) and RCP 6 (lower) are shown aggregated using GWP_100_ = 28 (cyan), GWP* with only the flow properties included (orange), and GWP* with both stock and flow properties using *r* = 0.75 and *s* = 0.25 (purple). The temperature response relative to 1900 as modelled by FaIR, from the methane radiative forcings in the RCP database are shown on the right axis (black dashed). Annual methane emissions are shown in the dotted lines (inner left axis). The temperature axis scales with the cumulative emissions axis by 1.8 K TtC^−1^ or 0.49 K TtCO_2_^−1^, which is the approximate slope of Fig. 2 for the GWP* flow and stock (purple)

To demonstrate the improved warming-equivalence of GWP*, cumulative emissions (left axes) are shown alongside the corresponding warming in Fig. 1. The change in GMST calculated from the methane radiative forcing is shown as a time series (black dashed line, right axis) for RCP 2.6 (upper), RCP 4.5 (middle) and RCP 6 (lower). The cumulative CO_2_-e emissions calculated using GWP_100_ (cyan) show that there is no agreement between CO_2_-e emissions and warming when methane emissions are stable or in decline. CO_2_-we emissions calculated with GWP*, especially when both stock and flow properties are included (purple), show a clear improvement with the cumulative emissions matching the temperature response.

Orange lines show cumulative CO_2_-we emissions retaining only the flow term i.e., setting *r* = 1, *s* = 0 in equation 1, equivalent to the definition in ref.,^12^ which scales with smoothed annual emissions (dotted line, inner left axis). GWP* defined by only the change in rate of methane emissions (i.e., as originally defined) overestimates the cooling that would occur under decreasing methane emissions because it fails to take into account the century-timescale response to earlier methane emission increases.

**Fig. 2:**
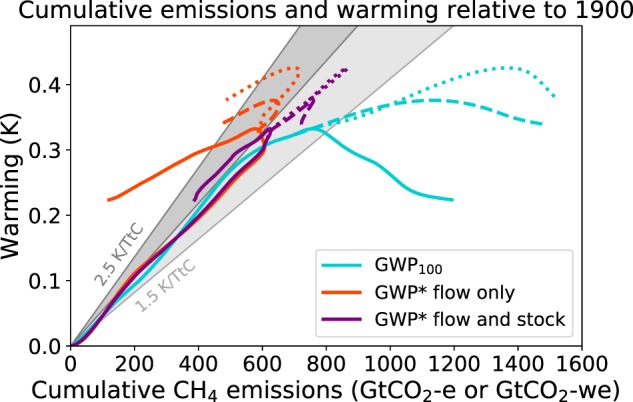
Cumulative methane emissions (1900–2100) from the historical period plus RCP 2.6 (solid), RCP 4.5 (dashed) and RCP 6 (dotted) converted to CO_2_-e emissions using GWP_100_ (cyan), GWP* using only the flow properties (orange), GWP* for both flow and stock properties using *r* = 0.75 and *s* = 0.25 (purple), against modelled warming response to the methane radiative forcing from the scenario database. TCRE values of 1.5 (shallowest gradient), 2.0, and 2.5 K per trillion tonnes Carbon equivalent (or 0.41, 0.55 and 0.68 K per trillion tonnes of CO_2_-we) are shown by grey lines

Figure 2 shows cumulative CO_2_-e and CO_2_-we emissions of methane plotted against the modelled temperature response, relative to 1900 and up to 2100, for the RCPs. The linear relationship between cumulative CO_2_ emissions and warming is the basis for the carbon budget concept, which describes how much CO_2_ can be emitted before any given threshold of global mean warming is reached. Previous studies ^26,27^ have computed CO_2_ budgets conditioned on specific scenarios for non-CO_2_ forcing. GWP* allows non-CO_2_ forcings to be included in the carbon budget itself, as it describes a linear relationship between cumulative CO_2_-we emissions of SLCPs and warming, as shown in purple in Fig. 2.

Under GWP_100_ (cyan) this relationship breaks down completely when SLCP emissions start to decline. Unphysically, GWP as traditionally used implies declining methane emission rates still contribute to increasing cumulative CO_2_-e, when they are in fact causing cooling. Hence, the negative gradient towards the end of the cyan scenarios in Fig. 2. If only the flow properties of methane are considered (orange), reducing methane emission rates are now equivalent to a negative CO_2_ emission, so the line ‘turns back’ on itself as cumulative emissions decline alongside reducing temperatures. Although much closer to a linear relationship than GWP_100_, in this exclusively rate-based version there is now more CO_2_-we removal than would be expected to explain a given amount of cooling, if we consider that the truly equivalent relationship should mimic that of CO_2_ emissions and temperature response, which is approximately linear.^28^ This is because the rate-based formulation alone does not account for the ongoing warming from the thermal equilibration to past methane emission increases. Incorporating a term that accounts for this behaviour, as proposed above (purple lines), largely overcomes the problem, providing a close match to a linear relationship for the scenarios tested here. The relationship shown here corresponds to a Transient Climate Response to cumulative carbon Emissions (TCRE, or the amount of warming per unit carbon emitted, shown by the grey lines in Fig. 2) of about 1.8 °C per TtC during the historical period and slightly less in the RCP projections. The slope changes because there is a time lag in the climate’s response, therefore negative emissions do not immediately reverse the effects of the same amount of emissions. This effect was reported by Zickfeld, et al.^29^ for CO_2_ removals. The TCRE depends directly on the prescribed TCR of the FaIR model used to generate the temperature: but, crucially, would be similar for all non-CO_2_ forcings provided their different efficacies are taken into account using effective radiative forcing.^1^ Adding a small correction to account for the fact that the climate system takes time to equilibrate to higher forcing permits a physically plausible interpretation of “equivalence” in the calculation of carbon budgets. This improves the accuracy of the temperature outcome of the equivalence metric, however it should be noted that this is still an application of GWP_100_ and therefore does not capture everything that a climate model includes. Collins, et al.^9^ investigate the impact of methane on the remaining carbon budget using an intermediate complexity climate-carbon model, and note that their results show a close correspondence to the GWP* metric as proposed in Allen, et al.^18^

### Physical interpretation and justification

We can illustrate the physical interpretation of *r* and *s* values by considering some more idealised scenarios. Setting the left-hand side of equation 1 (CO_2_-we emissions) to zero, we are able to calculate the methane trend required to be equivalent to no further CO_2_ emissions: Δ*E*_SLCP_/Δ*t* = −[*s*/(*rH*)]*E*_*SLCP*_, which is required to generate a radiative forcing pathway that will approximately stabilise temperatures over the time period Δt. Hence with *r* = 0.75, *s* = 0.25 and *H* = 100 years, [*s*/(*rH*)] = 0.3% is the rate at which methane emissions need to decline to give stable methane-induced warming. This makes zero CO_2_-we emissions under GWP* consistent with stable temperatures, matching the temperature response to zero CO_2_ emissions.

The definition of CO_2_-we using GWP* is independent of SLCP lifetime (assumed to be much shorter than *H*), but it does depend on the SLCP forcing history: if temperatures are close to equilibrium following a very gradual forcing increase over many centuries, a near-zero decline rate (near constant SLCP emissions) would be consistent with no further warming. Faster rates of decline would be required to maintain no further warming following a rapid increase in SLCP forcing, because the climate system would be further from equilibrium. Here we have based the coefficients and therefore the rate of decline on a combination of historical (1900 onwards) and future scenario emissions to encompass climate response in the near future to emissions over the last century.

We have used an empirical method to find a definition of GWP* that preserves the link between an emission and the warming it generates in the medium term up to 2100. The physical interpretation of equation 1 is that the flow term (with coefficient *r*) represents the fast climate response to a change in radiative forcing, generated by the atmospheric and ocean mixed-layer response.^30^ The timescale of this response is about 4 years here.^31^ The stock term (with coefficient *s*) represents the slower timescale climate response to a change in radiative forcing, due to the deep ocean response. This effect means that the climate responds slowly to past changes in radiative forcing, and is why the climate is currently far from equilibrium. We have approximated this response by treating a quarter of the climate response to a SLCP as “cumulative”. The timescale for this response is uncertain,^32^ and is of the order a few centuries, as discussed below.

The exponential decline of 0.3% per year corresponds to a time constant of about 300 years, consistent with the equilibration timescale of the climate system. This timescale is largely governed by deep ocean adjustment to relatively recent forcing increases identified by Geoffroy, et al.^32^ (multi-model mean of 290 years, with a standard deviation of 107 years). If the equilibration timescale of the climate system were shorter, then *s* would be lower. If the deep ocean response timescale were the same as the atmosphere and mixed ocean timescale (about 4 years), then *r* would be 1, *s* would be 0, and the definition of GWP* from Allen, et al.^12^ would not change.

The rate at which global methane emissions need to decline to reduce the rate of methane-induced warming to zero has not been explored systematically with complex models and would depend on the details of its atmospheric chemistry. An indication of the rate of decline of emissions of a generic SLCP required to stabilise SLCP-induced warming following a linear increase in SLCP emissions over a multi-decade period can be provided by considering the generic response to the following widely studied scenario: a linear increase in forcing to the equivalent of a CO_2_ doubling over 70 years, followed by constant forcing. If the thermal response of the climate system is characterised by a short (sub-decadal, atmosphere and mixed layer ocean) adjustment time, *d*_1_, and a long (multi-century, deep ocean) adjustment time, *d*_2_ ≫ 70 years, temperatures after year 70 adjust exponentially from their value at year 70 (the Transient Climate Response, or TCR) to their long-term equilibrium value (the Equilibrium Climate Sensitivity, or ECS) with an adjustment timescale d_2_. Hence, warming would rise at a fractional rate of (ECS−TCR)/(*d*_2_ × TCR) per year in the decades immediately after forcing is held constant (see supplementary Fig. 2). On multi-decade timescales longer than the SLCP lifetime, SLCP emissions would need to fall at the same fractional rate to yield no further warming, since the rate of SLCP emissions scales with SLCP-induced forcing and the temperature response is linear in forcing.

For representative values, (ECS = 2.75 °C, TCR = 1.6 °C, *d*_2_ = 239 years, after Millar, et al.^31^) this indicates a decline rate, (ECS−TCR)/(*d*_2_ × TCR), of 0.3% per year, corresponding to a time-scale of 333 years (the inverse of 0.3%/year). This is consistent with our estimates of *r* = 0.75 and *s* = 0.25 for *H* = 100 years, which give a time-scale (*r*/*s*) × *H* of 300 years. This indicates the approximate rate of decline of methane emissions required for no further warming. However, this timescale depends on the multi-century response of the climate system and carbon cycle feedbacks, all of which are poorly constrained by available observations and modelling: targeted experiments varying methane emission growth and decline rates would give a more precise indication.

**Fig. 3:**
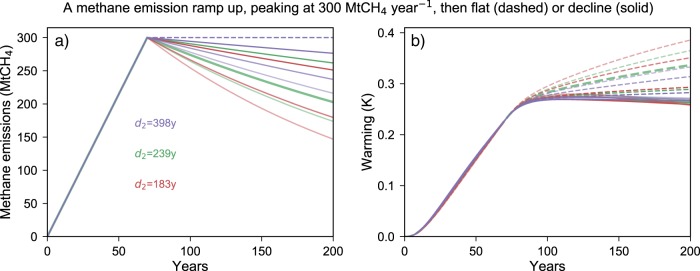
Demonstration of how different methane emission pathways **a** result in different long-term temperature responses **b** depending on the Equilibrium Climate sensitivity (ECS) and long-term climate adjustment time (*d*_2_). Dashed lines show methane emissions sustained at peak rates, while for solid lines methane emissions decline from peak at rates required to achieve approximately stable temperature responses under different climate model parametrisations. Colours represent different *d*_2_ values (1-sigma values of τ_s_ from Geoffroy, et al.^32^ in purple and red, and the default value from FaIR in green). Shading indicates different values of ECS, with the darkest shades showing ECS = 2.0 K, and lightest showing ECS = 3.2 K (lighter shading, i.e., greater ECS, requiring more rapid rates of decline in methane emissions **a** or resulting in increased long-term warming from sustained emissions **b**). Default values for FaIR are shown by the heavy green lines (ECS = 2.75 K, *d*_2_ = 239 years). See text for further details

The relationship between stable or declining methane emissions is shown in Fig. 3 for a range of model parameters in the simple climate model, FaIR (assuming a constant methane lifetime). Different colours show simulations with a 1-sigma range of *d*_2_ values from Geoffroy, et al.,^32^ and for a range of realised warming fractions (the ratio of TCR:ECS) based on that in the CMIP5 ensemble.^33^ The dashed lines show the scenario in which methane emissions are kept stable for 130 years after a 70-year ramp up to approximately present day emission rates. For all parameter combinations, constant methane emissions cause a continued warming. The solid lines show scenarios which reduce the emissions by a fractional rate of (ECS−TCR)/(*d*_2_ × TCR) per year to compensate for the slow climate response. As predicted, these give stable temperatures over the decades following the emissions peak. Decline rates range from between 0.06% (TCR:1.6 °C, ECS:2.0 °C, *d*_2_:397.0 years) and 0.55% (TCR:1.6 °C, ECS:3.2 °C *d*_2_:183.0 years). Note that these values have been calculated based on a simple climate model that emulates the response from complex climate models. There remains considerable uncertainty in how the real climate would evolve, for example through feedbacks that are not yet included in climate models, which may not be fully reflected in this range of estimates.

While methane emissions may thus appear to have a cumulative impact on global temperature, this is better interpreted as the delayed response to relatively recent methane emissions increases. Constant anthropogenic methane emissions, if maintained indefinitely, clearly have no further warming impact (being indistinguishable from constant natural emissions). This apparent cumulative impact is important, and captures the potential benefits of early methane mitigation^9^ not apparent through a solely rate-based equivalence, but is only about 25% (*s*) of the impact indicated by GWP_100_ and closer to that indicated by the 100-year Global Temperature-change Potential (GTP) including carbon cycle feedbacks. This should not, however, be interpreted as simple support for a lower metric value than GWP_100_: most scenarios and policy interventions involve changes in methane emission rates outside the range zero to −0.3%/year, in which case the first term on the RHS of equation 1 (neglected by conventional metrics) dominates.

## Discussion

We have demonstrated how it is possible to represent both the short-lived nature of methane and the long-timescale adjustment of the climate system in a single metric, GWP*. This metric allows SLCP emissions to be converted to CO_2_-equivalent emissions and preserve an unambiguous link to global warming, which we have therefore termed CO_2_-warming-equivalent. Just as many different methods have been proposed for the calculation of CO_2_-equivalent emissions, so there are multiple ways of calculating CO_2_-warming-equivalent emissions. Some, like GWP*, rely heavily on linearization; others, like CO_2_-forcing-equivalent emissions^19^ or explicit modelling of the temperature response,^9^ have a stronger physical justification and are likely to be more accurate for specific applications, at the cost of simplicity and generality. While complete disambiguation requires all details to be specified, the aim of CO_2_-we emissions is clear: to calculate the CO_2_ emission pathway that would yield the same global temperature change on all relevant timescales as that caused by the time-history of some non-CO_2_ climate forcer. Given this objective, appropriate methodological decisions should always yield broadly similar CO_2_-we pathways for the same climate forcer, in stark contrast to CO_2_-e emissions, for which legitimate methodological decisions such as the choice of time-horizon can change results by over an order of magnitude. Conventional pulse-based metrics treat SLCPs like methane as a stock pollutant only, thereby neglecting the rapid climate response to changes in SLCP emission rates, which dominate the temperature response while emission rates are changing.

Single-number metrics like GWP typically overestimate the cumulative effects of SLCPs; but there is some *apparent* cumulative impact of SLCP emissions, which arises not because they accumulate in the atmosphere, but because a component of the climate system’s response to past forcing increases is characterised by a slow equilibration timescale. Based on historical emissions and RCPs 2.6, 4.5 and 6, GWP* is found to best represent the temperature impacts of methane emissions by modifying the definition in Allen, et al.^12^ to weight the flow response (impact of changing methane emission rates) by 0.75 and the stock response (equilibration of the climate system to past methane emission increases) by 0.25.

The benefits of GWP* are most apparent when SLCP emission rates are declining, as this is when CO_2_-e emissions derived from conventional GWP_100_ would indicate a temperature response of the wrong sign (further warming instead of cooling). Under the Paris Agreement, nations have agreed to limit global warming to well below 2 °C, and to pursue efforts to limit it to 1.5 °C. Using GWP* to calculate CO_2_-we emissions can therefore be useful in linking emissions scenarios with temperature goals. It uses GWP_100_ in a novel way, and is thus consistent with current requirements that countries use this metric in emissions accounting. GWP* allows the contributions of all climate forcing agents to be aggregated to reach a global total cumulative CO_2_-we emission, which can then be multiplied by the TCRE to give an estimate of resultant warming over any given time period: Δ*T* = TCRE × [∑CO_2_−we + ∑CO_2_−*e*], where TCRE is the Transient Climate Response to cumulative carbon Emissions, ∑CO_2_−*we* is the cumulative short-lived GHG emissions aggregated using GWP* and ∑CO_2_−*e* is the cumulative long-lived GHG emissions aggregated using GWP_100_. This method provides a simple and transparent mechanism by which to estimate whether countries are on track to meet the Paris Agreement goals in the global stocktake. It also allows SLCPs and cumulative gases to continue to be included together in reporting mitigation ambitions, maintaining fungibility while improving environmental integrity.

## Methods

### Method to derive r and s

Data from the RCP database has been used to investigate how methane emissions relate to warming, and how different emission metrics represent that warming.

The RCP database contains emissions rates and radiative forcings for different greenhouse gases from 1765 to 2100 for the four RCP scenarios (RCP2.6, RCP4.5, RCP6 and RCP8.5). Here, the representation of methane based on GWP_100_ and GWP* is considered.

The methane emissions time series for each of the RCP scenarios are converted to CO_2_-e emissions timeseries using a GWP_100_ of 28.^1^ The temperature response to the methane radiative forcing (from the RCP database) is calculated using the FaIR model,^25^ with a factor of 1.65 applied to account for the secondary effects of ozone and stratospheric water vapour as recommended in Myhre, et al.^1^ Our analysis is consistent with assumptions in Myhre, et al.^1^ and therefore does not include more recent findings, for example updated radiative forcings from Etminan, et al.^34^ In all scenarios, the warming trend does not correspond closely to the cumulative CO_2_-e emissions calculated using GWP_100_.

The coefficients to weight the long and short term effects are found for each scenario using a linear regression model of the equation Δ*T* = *a*.*C*_GWP*_ + *b*.*C*_GWP100_, where *C*_GWP100_ is the cumulative CO_2_-e emission of methane defined conventionally using GWP_100_, and *C*_GWP*_ is the cumulative CO_2_-e emission of methane defined using GWP* from Allen, et al.^12^ with Δ*t* *=* 20 years. Coefficients *a* and *b* were found by ordinary least squares multiple linear regression using the statsmodels package in python (http://www.statsmodels.org), and then the normalised coefficients were set by defining *r* *=* *a*/(*a* + *b*) and *s* = *b*/(*a* + *b*), such that *r* + *s* = 1.

When radiative forcing is put into the FaIR model, a temperature response is calculated using an impulse response function as described in Smith, et al.,^25^ with 2 response timescales (239 and 4.1 years as default) and a default TCR and ECS of 1.6 and 2.75 K. The radiative forcings from the IPCC AR5 database used to drive this model have been calculated using the model MAGICC, which includes carbon cycle feedbacks. Note that this model does not include less well understood earth system feedbacks, such as permafrost feedbacks, which could act to accelerate warming. Therefore, the method is most suited for use in more ambitious mitigation scenarios where warming is slower, and abrupt positive feedbacks less likely to be triggered.

Table 1 shows the values for *r* and *s* that are generated using the above method, for historical data from 1900 and RCP 2.6, 4.5 and 6 data to 2100. The mean and standard deviation of these values are used in this work for *r* and *s*. The time period 1900 to 2100 is chosen as it represents the recent historical increase in methane emissions, as well as capturing three possible futures.

**Table 1:** Calculated values of *r* and *s*, based on 1900 to 2100 historical and RCP 2.6, 4.5 and 6 scenario data

Scenario	*r*	*s*
RCP 2.6	0.68	0.32
RCP 4.5	0.80	0.20
RCP 6	0.77	0.23
Mean	0.75 (0.05)	0.25 (0.05)

Standard deviation on the mean of the three scenarios is shown in brackets

## Supplementary information

Supplementary information

## Data Availability

The RCP datasets analysed during the current study are available at http://www.iiasa.ac.at/web-apps/tnt/RcpDb.

## Supplementary information

**Supplementary information** accompanies the paper on the *npj Climate and Atmospheric Science* website (10.1038/s41612-019-0086-4).

## Code availability

The code used to calculate *r* and *s*, CO_2_-we emissions and produce Figs 1 and 2 is available at https://gitlab.ouce.ox.ac.uk/OMP_climate_pollutants/co2-warming-equivalence.
