# Supplementary material for: Improved calculation of warming-equivalent emissions for short-lived climate pollutants
Source: NPJ Clim Atmos Sci. 2019 Sep 4;2(1):29. doi: 10.1038/s41612-019-0086-4 (PMC6814445; doi:10.1038/s41612-019-0086-4)
Supplement: Supplementary file 1 — Supplementary information [file 41612_2019_86_MOESM1_ESM.pdf]

Supplementary Information for Improved calculation of warming-equivalent emissions for short lived climate pollutants

Michelle Cain, John Lynch, Myles R. Allen, Jan S. Fuglestvedt, David J. Frame, Adrian H Macey

Supplementary Note 1: About Supplementary Figure 1

This is a reproduction of fig 2 from Allen, et al. [12] using r=0.75 and s=0.25 for GWP*. Note that, for consistency with Allen, et al. [12], warming is relative to pre-industrial temperatures, in contrast to fig 1 of this paper, where warming is shown relative to 1900. CO_2, methane and nitrous oxide emissions and forcing timeseries are from the RCP2.6 timeseries[24], with methane forcing scaled by 1.65 to account for the impact of methane on tropospheric ozone and stratospheric water vapour (Myhre et al[1], 8.SM.11.3.2) and nitrous oxide forcing scaled by 0.93 to account for interaction with methane (Myhre et al[1], 8.SM.11.3.2). 'Aerosol & other' represents the radiative forcing from aerosols and other minor climate forcers including industrial gases, and is derived directly from the total anthropogenic radiative forcing minus the contribution from CO_2, methane and nitrous oxide, after these adjustments, scaled to reproduce the AR5 estimate of total anthropogenic forcing in 2011. For GWP_{100}, a CO_2-equivalent quantity is generated by dividing this forcing by the Absolute Global Warming Potential $AGWP_H$ of CO_2 with a time horizon H of 100 years: hence modelling aerosols as generic climate forcers with arbitrarily short lifetimes. For GWP*, the CO_2-warming-equivalent quantity is generated by a modified version of Eqn. 1 calculated directly from radiative forcing, following Allen *et al.*[12]:

$$E_{\mathrm{CO_2\text{-}we}} = \frac{1}{\mathrm{AGWP}_{H(CO_2)}} \times \left[r \times \frac{\Delta F}{\Delta t} \times H + s \times F \right]$$

where ΔF is the change in forcing over the preceding Δt time-interval (here 20 years) and F the forcing in the current year, again with a time horizon H of 100-years. The coefficients r and s were set as 0.75 and 0.25, respectively. These are appropriate for modelling the impact of net aerosol forcing since, as the figure shows, this has a similar time-history to that of methane, with the opposite sign. As noted in the main text, further work may be required to confirm whether some individual climate forcers may be better modelled using alternative values.

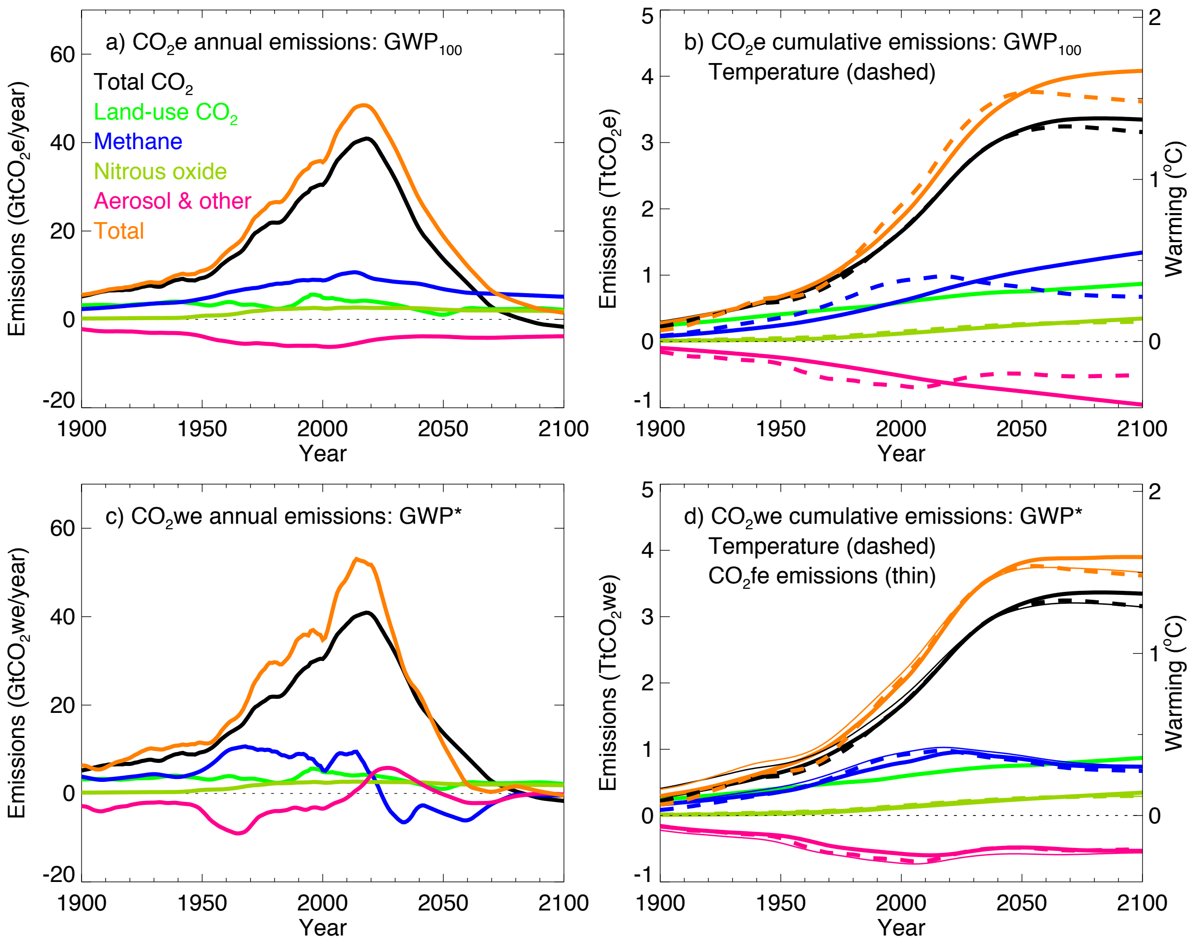

Supplementary Figure 1: Annual (a, c) and cumulative (b, d) CO_2-e and CO_2-we emissions under the GWP_{100} (a, b) and GWP* (c, d) metrics using historical emissions to 2015 extended with the RCP2.6 scenario. Dashed lines show global mean surface temperature (GMST) response to radiative forcings associated with these emissions (not available separately for land-use CO_2). Colors indicate gases following the legend in (a). Thin solid lines in d show cumulative CO_2-forcing-equivalent emissions closely tracking GMST response.

Supplementary Note 2: About Supplementary Figure 2

This figure illustrates a simple example of how CO_2 emissions (panel a) relate to CO_2 concentrations (panel b), radiative forcing (panel c) and global mean temperature change (panel d), explaining the origin of the fractional decay rate discussed in the text. The solid line shows the emissions required for a 1% per year increase in concentrations of CO_2 over 70 years, followed by stable concentrations, which means a linear increase followed by stable radiative forcing. This scenario leads to temperatures increasing at a fractional rate of $(\mathrm{ECS} - \mathrm{TCR})/(d_2 \times \mathrm{TCR})$ after year 70 shown by the dotted line in panel d, because of the slow timescale of the deep ocean climate response. This response characterises the difference between Transient Climate Response (the warming at the point of reaching doubled CO_2 concentrations under this scenario) and Equilibrium Climate Sensitivity (which is the warming after the climate is allowed to reach equilibrium following the same scenario). Steady methane emissions will lead to steady methane concentrations in the atmosphere (and therefore radiative forcing), assuming no other changes to the system, equivalent to the solid line.

In order to generate stable temperatures after year 70, CO_2 emissions must fall to zero, as shown by the dashed line. This leads to declining concentrations in the atmosphere and radiative forcing declining at a fractional rate of $(\mathrm{ECS} - \mathrm{TCR})/(d_2 \times \mathrm{TCR})$ shown by the dotted line in panel c. For methane emissions to produce this concentration/radiative forcing/temperature pathway over decade to century timescales, methane emissions must decline at a similar rate, which is 0.3% per year in the default configuration of our model.

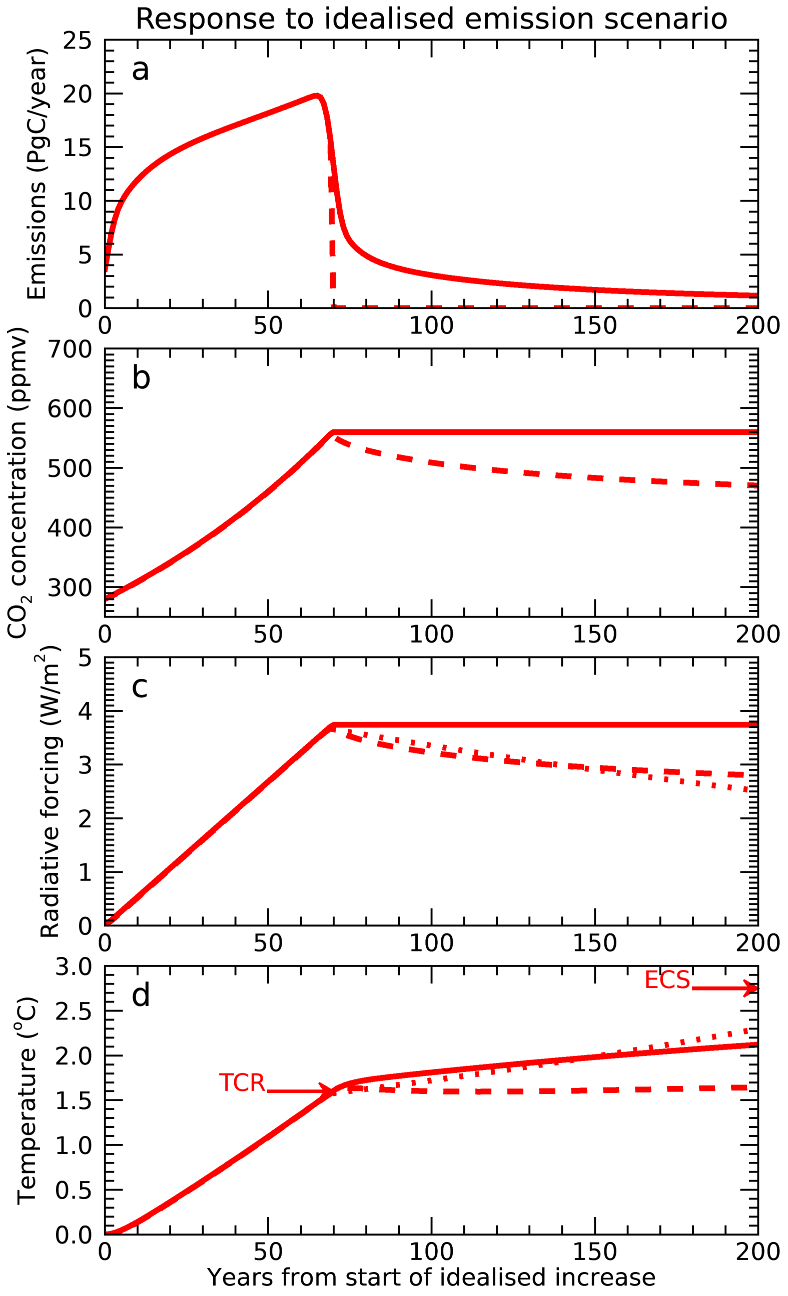

Supplementary Figure 2: Emissions (a), CO_2 concentrations (b), radiative forcing (c) and global temperature response (d) to two idealised CO_2 emissions pathways. Solid lines show emissions calculated to provide a 1% per year increase in CO_2 concentrations over 70 years, followed by stable concentrations thereafter. Dashed lines show the response to identical emissions up to year 70, followed by zero emissions thereafter. Dotted line in panel (d) shows a 0.3% per year increase in temperature after year 70 in response to constant CO_2 concentrations. Dotted line in panel (c) shows a 0.3% per year decline in radiative forcing consistent with zero emissions and constant temperatures. Calculated with the FaIR simple climate model with ECS=2.75 K and TCR=1.6 K and d_2=239 years.
